# Wound Healing Properties of Selected Natural Products

**DOI:** 10.3390/ijerph15112360

**Published:** 2018-10-25

**Authors:** Nurul ‘Izzah Ibrahim, Sok Kuan Wong, Isa Naina Mohamed, Norazlina Mohamed, Kok-Yong Chin, Soelaiman Ima-Nirwana, Ahmad Nazrun Shuid

**Affiliations:** Department of Pharmacology, Faculty of Medicine, Universiti Kebangsaan Malaysia Medical Centre, Jalan Yaacob Latif, Bandar Tun Razak, Cheras, Kuala Lumpur 56000, Malaysia; nurulizzah88@gmail.com (N.I.I.); jocylnwsk@gmail.com (S.K.W.); isanaina@yahoo.co.uk (I.N.M.); azlina@ppukm.ukm.edu.my (N.M.); gabrielchinky@gmail.com (K.-Y.C.); imasoel@ppukm.ukm.edu.my (S.I.-N.)

**Keywords:** antibacterial, anti-inflammatory, antioxidant, collagen, polyphenols, skin

## Abstract

Wound healing is a complex process of recovering the forms and functions of injured tissues. The process is tightly regulated by multiple growth factors and cytokines released at the wound site. Any alterations that disrupt the healing processes would worsen the tissue damage and prolong repair process. Various conditions may contribute to impaired wound healing, including infections, underlying diseases and medications. Numerous studies on the potential of natural products with anti-inflammatory, antioxidant, antibacterial and pro-collagen synthesis properties as wound healing agents have been performed. Their medicinal properties can be contributed by the content of bioactive phytochemical constituents such as alkaloids, essential oils, flavonoids, tannins, saponins, and phenolic compounds in the natural products. This review highlights the in vitro, in vivo and clinical studies on wound healing promotions by the selected natural products and the mechanisms involved.

## 1. Introduction

Skin is the largest organ of the body which makes up the outer covering of the living body. It consists of multiple layers of the ectodermal tissues guarding the underlying muscles, bones, ligaments, and internal organs. It also provides protection against microbes, heat, light and injury [1,2]. A wound is created when an injury to the normal structure and function of the skin occurs [3]. Wound healing refers to the complex and multifactorial process in response to a disruption of the normal anatomical structure and function of a skin tissue. It is characterized by a series of events, that is, inflammation, cellular phase (granulation), narrowing of wound area (wound contraction), collagen deposition (collagen formation), epithelial covering (epithelialization), and scar remodeling (cicatrization). The smooth progression of all these events leads to a successful completion of wound healing, and restoration of the disrupted anatomical and functional state of the skin [4,5,6].

In general, wounds healing in an orderly complex process that consists of three overlapping phases; inflammatory reaction, proliferation, and remodeling. The inflammatory phase involves vascular responses characterized by exudation, blood coagulation and hemostasis. During this process, varieties of immune cells from the blood vessels are attracted to the wound lesion and secrete pro-inflammatory cytokines. The inflammatory cells, notably neutrophils, may produce large amounts of reactive oxygen species (ROS). The ROS are essential in protecting the body against developing infection but, when present in excess amount, can simultaneously damage the surrounding tissues. In the normal process of wound healing, a reduction of immune cells and inflammatory cytokines should occur within a few days after an injury, and at the same time, migrating keratinocytes, fibroblasts, and endothelial cells start to secrete various growth factors. Subsequently, during the proliferative phase, there is a formation of the epithelium to cover the wound surface with concomitant growth of granulation tissue to fill the space of wound. The formation of granulation tissue involves proliferation of fibroblasts, deposition of collagens and other extracellular matrices (ECM) and development of new blood vessels (angiogenesis). The collagen synthesis causes contraction of wound and reduction of the wound size. The temporal ECM is gradually converted into a mature scar. During this process, type III collagen, which is a typical constituent in the granulation tissue, is replaced by type I collagen, a predominant constituent in the normal human dermis. This remodeling process will restore tissue structural integrity and functional competence. The process of wound healing is tightly regulated by multiple growth factors and cytokines released at the wound site [7].

Acute or normal wound healing proceeds through the orderly overlapping processes, allowing for repair of skin function and integrity in coordinated manner in healthy individuals. A normal wound healing usually occurs in 7 to 10 days [8]. Any alterations that disrupt the timely controlled healing processes would extend tissue damage and prolong the repair process, subsequently contributing to chronic wound healing [7,9]. Thus, a chronic wound healing can be defined as a disruption of the normal healing pathway. It can be caused by the underlying disease processes (such as diabetes or cardiovascular disease), infections, medications (steroids) and old age. The completion of full-thickness chronic wound healing may take months to years [8].

Wound healing process can be facilitated by natural products with medicinal properties. Many studies on the wound healing properties of natural products with anti-inflammatory, antioxidant, antibacterial and pro-collagen synthesis actions have been conducted. Their medicinal properties might be contributed by the bioactive phytochemical constituents of the various chemical families such as alkaloids, essential oils, flavonoids, tannins, terpenoids, saponins, and phenolic compounds [10]. Each bioactive agent may have specific function on wound healing properties. For instance, saponins can enhance the synthesis of pro-collagen [11], while tannins and flavonoids have antiseptic and antibacterial activities [12]. These phytochemicals can modulate one or more phases of the wound healing process. Furthermore, they are easily absorbed by the superficial layers of the skin [3]. Due to these properties, natural products and their phytochemicals have important roles in wound healing and they are used in the design of new synthetic compounds for this purpose [13]. This review aims to highlight the use of selected natural products (including *Curcuma longa*, vitamin E, honey and sea cucumber) in promoting wound healing and their mechanisms. These natural products have been used extensively in wound care management with excellent outcomes. The pre-clinical and clinical evidence studies of these natural products are also discussed in this review.

## 2. Curcuma longa

*Curcuma longa* (*C. longa*) or its common name, turmeric, is a member of the ginger family (*Zingiberaceae*). *C. longa* has been used since antiquity as a dye and condiment. In Asian countries, it is consumed daily for centuries and no toxicity has not been reported [14]. However, prolonged feeding of curcumin has been associated with incidences of ulcers, hyperplasia and inflammation of the forestomach, cecum and colon in male rats [15]. The bright yellow color of *C. longa* is derived primarily from the fat-soluble, polyphenolic pigments known as curcuminoids. Curcumin (diferuloylmethane), the principal curcuminoid found in *C. longa*, is generally considered as its most active constituent. Other curcuminoids found in turmeric include bisdemethoxycurcumin and demethoxycurcumin [16].

Several biological activities of curcumin have been identified, including anti-inflammatory, anticarcinogenic, anti-infectious, antioxidant, anti-apoptotic and wound healing activities [6,17,18,19,20,21]. To date, many in vitro [22,23,24] and in vivo [25,26,27] studies have been conducted to elucidate the effects of curcumin on wound healing activities. Despite the extensive in vitro and in vivo studies on wound healing activities of curcumin, there is a major barrier for wider clinical applications which reduces its therapeutic activity. The hydrophobic nature and photosensitive property of curcumin are the drawbacks for oral administration and topical application respectively. Curcumin possesses very poor aqueous solubility, permeability and bioavailability and is classified as a Biopharmaceutics Classification System (BCS) class IV molecule [28]. To enhance its bioavailability, curcumin has been incorporated into different formulations using hydrogel, nanoparticles, micelles, hyaluronic/oleic acid-loaded and glucosylation of the hydrophobic molecule in pre-clinical studies (Table 1). Curcumin has shown potential as a wound healing agent.

In these studies, curcumin was applied to the animals via topical dressing or given orally, as curcumin alone or incorporated into different formulations. In the study by Rao et al. (2015), they tested the effects of curcuminoids (curcumin, tetrahydrocurcumin (THC), and glucosyl-THC) topically on cutaneous excision-punch wound rat model [29]. Tetrahydrocurcumin is a major in vivo colorless hydrogenated metabolite of curcumin, while glucosyl-THC is a product of glucosylation of the hydrophobic molecule. In the study, they have proven that at 2% concentration of the curcuminoids, glucosyl-THC showed the best result in wound healing, with complete healing at 21 days. This indicated that glucosylation of the hydrophobic molecule may improve the bioavailability and pharmacological activities of curcumin [30,31]. Apart from glucosylated curcumin, in a study by Mohanty et al. (2012), curcumin formulated into oleic acid polymeric bandage (COP) resulted into increased wound reduction and enhanced cell proliferation [32]. The use of COP enables sustained release of curcumin in solubilized form and the sustained availability of solubilized curcumin metabolites (sulfates and glucoronates) at the wound site, which may promote better healing.

Other than that, a study by El-Rafaie et al. (2015) showed that healing efficacy of hyaluronic acid-based nanocarrier gel core incorporating curcumin in burn wounds of rats [33]. While, a study by Sharma et al. (2018) showed that the conjugated curcumin with hyaluronic acid (HA)-treated animals had better healing as compared to treatment with HA-free curcumin and HA alone [34]. Dai et al. (2017) have optimized a carrier system that enhanced solubility and availability of curcumin by embedding it into gelatin-based electrospun nanofibrous mats [35]. The application of this curcumin/gelatin-blended nanofibrous mats (NMs) enhanced regenerative process in rat model of acute wounds and thus may provide a method for translating this ancient medicine for use in modern wound therapy. Studies have shown that combination of curcumin with chemicals that possess excellent water absorption and fluid affinity such as oleic acid, hyaluronic acid and gelatin, improved bioavailability of the poorly soluble curcumin and thus promote better wound healing. Jagetia and Rajanikant (2004) performed an intervention in vivo study by pre-treating healthy mice orally with 100 mg/kg of curcumin prior to whole body γ radiation and induction of full-thickness wound. The pre-treatment with curcumin significantly enhanced the rate of wound contraction, decreased mean wound healing time, increased synthesis of collagen, hexosamine, DNA, and nitric oxide that was impaired due to radiation [36].

Enhanced wound healing activities such as good epidermal regeneration, dense granulation tissue and improved collagen content were observed in animals with wound impairment induced by streptozotocin, dexamethasone and radiation [26,34,36,37]. In a study by Kant et al. (2014), curcumin incorporated with PF-127 hydrogel (25%) was topically applied to impaired wound of diabetic model. The wound contraction rate and the expressions of inflammatory cytokines/enzymes were increased, indicating enhanced wound healing [37]. In addition, a recent study by Yen et al. (2018) has also reported accelerated wound healing using similar type of hydrogel with curcumin but with some modifications on the formulation applied topically to healthy C57BL/6J male mice [25].

The wound healing effects of curcumin may be contributed by its anti-oxidant and anti-inflammatory properties. Kant et al. (2014) showed that curcumin applied topically on skin wound showed decreased expressions of tumor necrosis factor-alpha (TNF-α), interleukin-1 1 beta (IL-1β) and matrix metalloproteinase-9 (MMP-9) [37]. Additionally, ROS is a crucial regulator of wound healing process and is required at low levels for defense against invading pathogens, and to mediate intracellular signaling [38,39,40,41]. However, excessive amounts are produced in wounded and inflamed tissue by NADPH oxidase, an enzyme complex, which is expressed at particular high levels by inflammatory cells [42,43]. Curcumin treatment was shown to decrease the production of ROS and increase cellular proliferation in accelerating wound healing [20]. Curcumin application on the wounded skin showed increased levels of anti-inflammatory cytokine including interleukin-10 (IL-10) and antioxidant enzymes that is, superoxide dismutase (SOD), catalase (CAT) and glutathione peroxidase (GPx) [37].

Transforming growth factor-beta (TGF-β) and nitric oxide (NO) are important in wound healing process. In previous study by Mani et al. (2002), curcumin embedded with polyethylene glycol base significantly accelerated wound healing and enhanced expression of TGF-β1 and its receptors type I TGF-β (*tIrc*) in both normal and impaired healing [26]. During wound repair, TGF-β1 may stimulate epidermal keratinocytes to express integrins, which facilitate the migratory component of reepithelialisation [44]. The greater expression of TGF-β1 in curcumin-treated wounds could therefore be a basis for the enhancement of healing by curcumin. Nitric oxide production is regulated by inducible nitric oxide synthase (iNOS) that is produced by infiltrating inflammatory cells. A study by Thornton et al. (1998) suggested that wound nitric oxide enhanced collagen accumulation [45]. Indeed, a study by Yen et al. (2018) revealed that the levels of collagen were significantly higher in curcumin-treated wounds than control group, resulting into increased wound tensile strength [25].

For an effective use of curcumin as wound healing agent, it can be incorporated into a patch for prolonged release of the substance. Apart from glucosylation, there may be other ways to chemically modify its structures to make it more water soluble. Cucurmin also has antimicrobial activity, hence it may be used in infected wounds. However, it is unclear about the bioavailability of curcumin through topical route and whether it will cause hypersensitivity. There are also aesthetics issues such as yellow staining of the skin and clothes and some might not like its smell. In summary, curcumin could be a promising therapeutic agent for wound healing via multiple biological actions such as regulating anti-oxidant and anti-inflammatory mediators as well as promoting collagen deposition at the wound site. As there are many pre-clinical studies using different formulations of curcumin to improve its bioavailability, further investigations are recommended to examine its effects in clinical trials.

## 3. Vitamin E

Vitamin E consists of two major subfamilies, namely tocopherol and tocotrienol. Both tocopherol and tocotrienol exist in alpha (α), beta (β), gamma (γ), and delta (δ) forms, depending on the position of the methyl groups on the chromanol ring [46,47]. The main sources of vitamin E include wheat germ oil, wheat, rice bran, barley, oats, coconut, palm and annatto [46]. Since the discovery of vitamin E as a potent lipophilic anti-oxidant, anti-inflammation and platelet aggregation inhibitor [48], it has been regarded as a potential agent in treating burns, surgical wound and other wounds. Vitamin E acts as a reducing agent by scavenging free-radicals to prevent oxidative reaction that can cause tissue damage [49]. In an earlier in vivo study, Shukla et al. (1997) reported that wounding resulted in the loss of both enzymatic [SOD, GPx, CAT, glutathione-S-transferase (GST)] and non-enzymatic (ascorbic acid, vitamin E, and glutathione) antioxidants, which were partially or completely recovered following healing. Findings obtained from this study suggested the utilization of free radical scavengers to facilitate the self-healing of a wound [50].

Most preclinical studies reported promising wound healing properties of vitamin E using healthy murine, healthy swine, and diabetic murine models (Table 2). In these studies, vitamin E was administered to the animals via topical dressing, oral gavage or injections [intramuscularly (i.m.) or intraperitoneally (i.p.)]. Accelerated wound closure rate was detected in both genetically and chemically-induced diabetic rodents [51,52,53,54,55]. Vitamin E-related intervention facilitated the formations of new blood capillaries (angiogenesis), epithelium (epithelialization), tissue granulation, collagen fibers and dermis layers at the excisional and incisional wounds. In retrospect, Simon et al. (1994) found that pre-treatment of vitamin E via injection or dressing decreased the wound healing time for laser injury in healthy female Yorkshire pig by approximately one week compared to the negative control group [56]. In addition, a recent research fabricated a novel organic-inorganic dressing of copper-doped borate bioactive glass/poly(lactic-co-glycolic acid) loaded with vitamin E (3%), which has shown effectiveness in healing skin defects in the healthy rats [57]. Raxofelast, a synthetic analogue of vitamin E, was also found to restore wound healing to nearly normal levels in diabetes-impaired wound in mice [58]. Another in vivo study reported paradoxical outcomes whereby the animals with a 4 cm^2^ full-thickness incision treated topically with 0.1435 g of test formulation (containing medium chain triglycerides, linoleic acid, vitamin A, vitamin E and soy lecithin) did not show any acceleration in the process of tissue healing. In fact, the exact dose of vitamin E in the test solution used in this study was not mentioned [59].

Several human studies provided evidence that the interventions containing vitamin E (administered either orally or topically) conferred beneficial effects on wound healing. In a randomized, double-blind, placebo-controlled pilot study, a total of 32 children aged 2–15 years with thermal trauma [burn area more than 10% but less than 50% of total body surface area (TBSA)] were randomized into no supplementation or oral anti-oxidant supplementation groups. The anti-oxidant supplement consisted of a mixture of vitamin C (1.5 times upper intake level), vitamin E (1.35 times upper intake level) and zinc (2.0 times recommended dietary allowance). The findings showed that the supplemented group required less time to complete tissue repair in wounds [60]. In the subsequent year, a single-blinded prospective study was conducted to compare the pre- and post-surgery use of vitamin E (dose not mentioned) in the form of Lipogel^®^ on surgical incisions in children underwent elective inguinal surgery (n = 428). Topical application of vitamin E before and after surgery significantly improved surgical wound healing with no cases of wound infection and no development of keloids in all the patients [61]. Recently, another prospective study evaluated the effects of silicone-α-tocopherol acetate dressing (dose not mentioned) at skin graft donor sites in patients requiring split-thickness skin graft for any reconstructive purpose (n = 60, aged 9–82 years). Significant reductions in pain perception and total cost of treatment were observed following the intervention [62]. However, vitamin E seems to have no apparent effects in reducing scars. Researchers have conducted a prospective randomized, double-blind trial to assess the effects of 5% tocotrienol on hypertrophic scars in subjects with recently (less than 2 weeks) healed operational scars (n = 122; aged 16–59 years). Topical application of the prescription twice a day had no significant effect on the scar appearance and vascularity after 4 months [63]. A report by Baumann and Spencer (1999) revealed that there was no improvement in the cosmetic appearance of scars with high incidence of contact dermatitis in patients underwent skin cancer removal surgery (n = 12) after treated with Aquaphor^®^ mixed with two capsules of d-α-tocopheryl at a concentration of 320 IU/g [64].

The inflammation stage is the first event during the wound healing process. Neutrophils and macrophages are recruited to secrete inflammatory cytokines and ROS, thus providing the bacteriostatic effects against invading bacteria. Overwhelming inflammatory response and ROS production exceeding the capacity of the endogenous defense may lead to tissue damage, thus suggesting the pivotal role of inflammation and ROS modulation during wounding [65]. Previous studies indicated that vitamin E regulated oxidative stress [decreased 4-hydroxynoneal (4-HNE) and malondialdehyde (MDA) levels] and inflammation [reduced nuclear factor kappa-light-chain-enhancer of activated B cell (NF-κB), TNF-α, and IL-1β] in diabetic cutaneous wounds [53,54,58]. Vitamin E also increased the anti-oxidative enzymes such as SOD, GPx and CAT responsible for detoxifying ROS and oxidized macromolecules in wound tissues [52]. Indeed, vitamin E also possessed beneficial effects in the subsequent stages (proliferation and remodeling phases) of wound healing process by stimulating the formation of new blood vessel along with re-epithelialization, matrix deposition, and collagen synthesis [51,52,57,58]. Apart from orchestrating the wound healing process, vitamin E has been demonstrated to act synergistically with antibiotics (tigecycline or daptomycin) to exhibit anti-microbial effects in a healthy mouse model of wound infection caused by methicillin-resistant *Staphylococcus aureus* (MRSA). Vitamin E and antibiotic increased levels of natural killer cell cytotoxicity, wound repair markers, and leukocyte populations in mice [66,67].

Vitamin E is lacking any clear mechanism apart from suppressing inflammation and oxidative stress. Therefore, more research is needed on tocotrienol. As a conclusion, vitamin E may be a potential therapeutic agent for wound healing via suppressing bacterial growth as well as excessive oxidative stress and inflammation. However, no appreciable effect of vitamin E on scar appearance has been reported. Further investigations are recommended to draw a better conclusion on the clinical utility for scar management.

## 4. Honey

Honey is a yellowish-brown, sweet and viscous sugar solution gathered from nectar of flowers by bees and other insects. For the bees, the floral nectar is bio-transformed, deposited, dehydrated, stored and left in the honeycomb to ripen and mature [68]. It contains a mixture of carbohydrates (80–85%), water (15–17%), protein (0.1–0.4%), ash (0.2%) with small quantities of enzymes, vitamins, minerals, amino acids and organic acids [69,70]. The use of honey as a remedy to treat wounds has been practiced in folklore. This traditional belief has been scientifically proven by pre-clinical studies and clinical trials whereby honey accelerated the healing of a multitude of wound injuries, including burns, surgical wounds, infected surgical wounds, and malignant wounds. Findings from in vivo studies showed that honey-dressed wounds generally healed faster compared to the untreated wound (Table 3).

The characterization of wound lesions indicated by improvements in area of epithelialization, angiogenesis, breaking strength, tissue granulation, collagen deposition, and wound contraction after honey treatment had been performed previously. Some studies reported that the efficiency and efficacy of honey in wound healing was comparable to other usual wound care products such as Intrasite Gel^TM^ [71], Alpha^®^ ointment [72] and hydrocolloid dressing [71] but more superior than hydrofiber silver and hydrofiber dressings [73]. The aesthetic outcome of honey treatment was also reported, as thinner scars were formed at wound site of the animals [71]. However, Nakajima and colleagues pinpointed that Japanese honey (Acacia, Buckwheat flour or Chinese milk vetch) exerted limited benefit as some wounds were not completely healed after 14 days, although the wound area was decreased in the inflammatory phase [74]. In subsequent year, another study was conducted to investigate the effects of the combined use of Japanese honey (Manuka, Acacia or Chinese milk vetch) and hydrocolloid dressing on wound healing. The results showed that this intervention did not enhance cutaneous wound healing, characterized by larger wound size, delayed re-epithelialization and collagen deposition in the wounds [75].

To our knowledge, all honeys are not the same as it differs in the chemical compositions, biological properties and medicinal value depending on the environmental factors, geographic area, and nourishments collected from the plants [69]. The dose, frequency, duration, and route of administration for honey are also different in the animal experimentations. These variations may attribute to the distinct outcomes observed in these studies.

Additionally, the effects of honey in wound healing have been validated in several human studies with different types of wound. In a pilot study, a total of 10 patients with cutaneous wounds (aged 10–70 years) were enrolled. Honey showed beneficial effects in developing healthy granulation (pink-colored tissue as an indicator of healing), reducing burning sensation, and discoloration surrounding the skin at day 7, as well as reducing pain, swelling, and tenderness at day 15 [76]. In a triple-blinded randomized prospective clinical trial involving 75 women with caesarean section [mean age: 27.70 ± 4.97 years (honey group), 26.57 ± 4.88 years (placebo group)], honey was found to reduce redness, edema, ecchymosis, discharge and approximation of wound edges (REEDA) score at day 7 and 14 [77]. Similar outcome was reported in nulliparous women with episiotomy incision (n = 120; aged 18–35 years) whereby honey significantly reduced the REEDA score. However, treatment with honey did not reduce pain intensity in episiotomy wounds [78]. The effects of honey on malignant wounds were also evaluated by Lund-Nielsen et al. (2011). In this randomized study, patients with advanced stage cancer and malignant wounds (n = 69; age: 47–90 years) were recruited [79]. The patients experienced a decrease in wound size, malodor and an improvement of wound cleanliness over time after using the honey-coated bandage. There were no significant changes for exudation and wound pain during the intervention period [79]. Another randomized clinical trial was performed to assess the wound healing properties and cosmetic outcomes of honey on surgical wounds in female patients underwent an elective plastic surgery (n = 52; aged 34 ± 3 years). The findings showed that the application of honey dressing reduced mean width of the scar and led to a lower probability of developing surgical complications such as marked erythema, dehiscence and infection [80]. Besides, the effects of honey on burn wounds have been evaluated in several studies. In the first study, patients with partial thickness burns (<30% TBSA) and requiring tangential excision (n = 10, age: 2–60 years) were included and dressed with Tualang honey. The bacterial load on wound was significantly lowered on day 6 onwards, suggesting that Tualang honey might have bactericidal and bacteriostatic effects [81]. In the second study, 84% and 100% of patients with superficial thermal burns (<40% TBSA) (n = 100; aged 3–70 years) showed satisfactory epithelialization in honey-dressed wounds after 7 and 21 days, respectively [82]. In patients with first and second degree of burns (<50% TBSA) (n = 108, aged 10–68 years), the duration of healing in honey-treated wounds was also shorter compared to silver sulfadiazine-treated wounds [83,84]. Overall, the positive outcomes of honey were consistently reported in the documented human studies.

The administration dose or amount of honey applied on wound was not mentioned in some of these studies. Only the anti-microbial activities of honey in wound were investigated. Evidence on other biological activities, particularly the immunomodulatory and anti-oxidative actions of honey, which have been proposed as the possible underlying mechanisms in wound healing enhancement are limited. The common characteristics of honey, including high sugar content (high osmolarity), high viscosity, low water content, and low pH (high acidity), provide a protective barrier against invading pathogens. Honey is not an appropriate medium for bacteria as it produces osmotic effect to dehydrate microbes thus hindering their growth. The ability of honey to produce hydrogen peroxide following dilution with water is another proposed reason for its bacteriostatic and bactericidal properties. This is with the exception of Manuka honey as it is a non-peroxide honey displaying anti-microbial effects [85]. Nonetheless, a variation in the anti-bacterial properties of honey was observed in laboratory and clinical studies. Data published by Khoo et al. (2010) showed that Tualang honey was superior in inhibiting the growth of *Pseudomonas aeruginosa*, but less effective in the control of *Acinetobacter baumannii* wounds as compared to hydrofiber silver and hydrofiber [73]. On the other hand, Tualang honey exhibited negligible effect in *Klebsiella pneumonia*-inoculated wound. Another report by Nasir et al. (2010) revealed that Tualang honey was not as effective as silver-based dressing for Gram-positive bacteria, such as *Streptococcus* spp., *Staphylococcus aureus*, and coagulase-negative *S. aureus* [81].

The immunomodulatory and anti-oxidative effects of honey have been previously established in in vitro wound healing model or other animal models. In vitro studies found that incubation of honey and monocytic cells stimulated the productions of TNF-α, IL-6, and IL-1β from monocytes, thus speculating the therapeutic potential of honey in wound might be elicited through the stimulation of pro-inflammatory cytokines [86,87]. In contrast with the immunostimulatory effects, the anti-inflammatory properties of honey have also been elucidated. Honey has been shown to attenuate the nuclear translocation and activation of NF-κB with subsequent decrease in inflammatory mediators such as cyclooxygenase-2 (COX-2) and TNF-α [88]. Another study by Sell et al. (2012) indicated that honey modulated the release of pro- (TNF-α) and anti-inflammatory cytokines (IL-10) as well as stimulated proliferation, collagen matrix production and migration of fibroblasts into in vitro wound healing model [89]. Moreover, honey has anti-oxidative ability to neutralize oxidative stress by increasing the levels of GPx, GSH, SOD, and NO as well as decreasing the levels of MDA, TNF-α, IL-6 and IL-1β in animals [90]. Taken together, it has been postulated that honey may be a potent immunomodulator (with both pro- and anti-inflammatory activities) and anti-oxidant at different stages of wound healing processes.

In conclusion, it is believed that honey may appear as an alternative and affordable approach for wound management via eradication of bacterial infection. Even though the anti-microbial efficacy of honey varies on different microorganisms, microbial resistance to honey has never been reported [91]. Honey is also less sticky to the wound base leading to easier wound dressing, has no allergic reaction and side effects [73,92]. Indeed, further scientific proof from both in vivo studies and clinical trials are appreciated to support the notion that honey is effective in enhancing wound healing via its immunomodulatory and anti-oxidative abilities. However, it is difficult to standardize honey composition due to its variety. There are also concerns of its high sugar content to diabetic patients, fake honey and sustainability of honey harvesting.

## 5. Sea Cucumbers

Sea cucumbers are soft and cylindrical-bodied echinoderms that used their tentacles for feeding microscopic algae, absorbing nutrients from the organic matter [106,107]. They are commonly known as *trepang* (Indonesia), *beche-de-mer* (France), or *gamat* (Malaysia) are marine invertebrates which have been utilized as a culinary delicacy, particularly in some parts of Asia [108,109]. The Chinese regard sea cucumbers as the most nutritious and luxurious seafood. They are used to treat several conditions including constipation, problems related to the kidneys, and reproductive disorders. Sea cucumbers contain high protein levels, low sugar and fat content, but no cholesterol [110].

Sea cucumbers are unique for their capability to renew or regenerate themselves and to repair as well as to restore lost organs. Some of the species can also divide themselves into two halves and each half grows and survives as a new creature. There are approximately 1500 species of sea cucumbers worldwide with the greatest number found in the Asia Pacific Region. In Malaysia, there are almost 42 species of sea cucumbers identified and *Stichopus* species was the most harvested for their medicinal properties [111]. Among the identified species, *Stichopus hermanni* species commonly known as *gamat emas* was believed to be the most effective species to treat joint pain, wound injuries, and back pain traditionally.

Dried or extracted sea cucumbers are commercially marketed as sea cucumber extracts and are frequently blended with well-known herbs to produce medicinal products in the form of nutritional supplement, either in capsules or tablets, ointment, toothpaste, body lotion, and facial cleanser. The supplements can be taken orally while the ointments can be applied to the face, gum, mouth, hands, feet, and other body parts [112]. They are well recognized as tonic and traditional remedy especially in East and South-East Asia. The content of bioactive compounds extracted from different sea cucumber species, especially triterpene glycosides (saponins) [113], chondroitin sulphates [114], glycosaminoglycans [115], sulphated polysaccharides [116], sterols (glycosides and sulphates) [117], phenolics [118], peptides [119], cereberosides [120] and lectins [121] might contribute to medicinal benefits and health functions of sea cucumbers.

Sea cucumbers have long been recognized in the folk medicine due to their high nutritious value, as they could nourish the body, moisten the intestine, tonify kidney, as well as treat stomach ulcers, asthma, hypertension, rheumatism, pain, gout, asthma, eczema, hyperglycemia, hypertension and wound healing [122,123]. Several pre-clinical have validated the wound healing properties of sea cucumber. Findings from in vivo studies revealed that wounds treated with sea cucumber generally demonstrated enhanced healing compared to the untreated wounds in various types of animal models (Table 4).

In these studies, accelerated wound contraction rate was observed with topical application of several sea cucumber species extracts on wounds induced in animals [124,125,126,127]. The process of wound contraction is an imperative event in wound healing phase because it acts as a major contributor to the healing of a full thickness wound [128]. This was supported by a significant improvement in contraction rate of full thickness excisional wounds in rats treated with topically applied sea cucumber extracts [124,125].

Apart from direct topical application, Zohdi et al. (2011) reported a significant wound contraction rates for *S. hermanii*-based hydrogel wound dressing at day 21 and 28 post burn wound. However, no significant differences were detected at day 7 and 14 post burn wound. This may indicate that the novel cross-linked gamat hydrogel (*S. hermanii)* dressings act at later stage of wound healing phase, which might be associated with reservoir capacities of hydrogel for the delivery of “*gamat*” on the wounded skin. The development of a sustained and controlled release system would immobilize biologically active species for longer period in hydrogel matrices. This would substantially increase the utility of incorporated sea cucumber during tissue repair and effectively interact with the wounds, thus facilitating healing process at later stage [127]. In contrast, Subramaniam et al. (2013) showed that topical application of *S. horrens* exhibited significantly smaller wounds at day 4 (*p* < 0.001), but no significant change on days 8, 12, 16 and 21 when compared to negative control (*p* > 0.05). This indicated that direct topical application of sea cucumber may enhance wound contraction at the initial period of wound healing [125]. However, in comparison with *gamat*-based hydrogel, the use of different species might contribute to the variations of wound contraction rate at different period.

In addition, the wound healing properties of another sea cucumber species, *Stichopus choronotus* could also be observed at the initial phase of wound healing. Mazliadiyana et al. (2017) reported that the intermediate dose, 0.5% *w*/*w* aqueous extract of *S. Chloronotus* emulsifying ointment mixture demonstrated the highest percentage of wound reduction compared to other groups (0.1%, 1.0%, positive and negative controls) from day 6 following wound creation. At optimum dose of *S. Choronotus*, healing of minor wound was significantly better than the gold standard (flavine—a yellow acridine dye with antiseptic property) [126]. According to Althunibat et al. (2009), aqueous extract of *S. chloronotus* showed superior antioxidant activity by approximately 80% compared to its organic extract [129]. Besides that, a study by Fredalina et al. (1999) on fatty acids composition of *S. chloronotus* showed higher content of docosahexaenoic acid (DHA) polyunsaturated fatty acids (PUFA) in aqueous extract compared to its organic extracts [130]. The marine-derived DHA PUFA may increase pro-inflammatory cytokine production at wound sites assisting to control infection and preparing tissue for further repair by enhancing phagocytic activity, stimulating keratinocyte migration at wound edges, fibroblast chemotaxis and proliferation, breakdown of extracellular matrix proteins and regulating the release of additional cytokines and growth factors [131,132]. The method of sea cucumber extraction is an important matter, as different methods of extraction may produce different percentage of bioactive compounds. This was supported in the study by Mariana et al. (2011), whereby direct topical application of *S. badionotus* methanolic extract showed no significant wound reduction compared to vancomycin on the healing of heat-burn wound inoculated with MRSA [133]. Another factor that may contribute to the successful healing properties is the use of aqueous extract with an emulsifying ointment, which will retain the treatment time of the water-based crude extracts. Hence, there might be an increased rate of wound reduction if the water-based crude extracts were applied locally with an oil base or cream.

Similar with the study by Mazliadiyana et al. (2017) [126], Arundina et al. (2015) reported that *S. hermanii* promoted the initial phase of wound healing of traumatic ulcer in rats [134]. In this study, the treatment was applied one time to ulcer and the rats were sacrificed on the 4th day to examine the number of lymphocytes. The intermediate dose of 40% aqueous extract of *S. hermanii* showed the largest number of lymphocytes, compared to other groups (20%, 80% and controls). To contradict, the highest dose (80% aqueous extract) of *S. hermanii* had lower number of lymphocytes [134]. Thus, appropriate dose should be applied, as different concentrations of extract may act differently. Lymphocytes play an important role during inflammation by releasing lymphokines which affect the number of other inflammatory cells. One of the lymphokines produced is interleukin-2 (IL-2), which can bind to heparan sulphate and help the proliferation process of T-lymphocytes. T-lymphocytes will then secrete TGF-β, stimulating fibroblast proliferation which plays a role on wound healing [135].

As being discussed, the mechanisms that might contribute to the wound healing property of sea cucumber includes anti-inflammatory [127] and antibacterial properties [133]. The anti-inflammatory effect of sea cucumbers in clinical settings was studied by Haryanto et al. (2017) [136]. In this prospective observational study, sea cucumbers incorporated into Carbopol^®^ gel base were applied topically to diabetic foot ulcer patients for 12 weeks. The results showed significant differences in the TNF-α level between weeks 0 versus 8, 0 versus 10, and 0 versus 12 in the sea cucumber group (*p* = 0.005, *p* = 0.006, and *p* = 0.010) respectively. The content of saponin in sea cucumber extract may prevent the lipopolysaccharide-induced production of TNF-α by NF-κB. NF-κB is a transcription factor that regulates the transcription of many genes associated with inflammation [137]. Moreover, the major fatty acids in sea cucumber; eicosapentaenoic acid (EPA) and DHA may stimulate the production of resolvins and protectins (anti-inflammatory mediators) through cyclooxygenase (COX-2) and lipoxygenase (5-LOX) pathways. Resolvins primarily inhibit IL-1β production, whereas protectin inhibits TNF and IL-1β production [138].

As for antibacterial property of sea cucumber, Mariana et al. (2011) concluded that sea cucumber crude extract has potential to become an alternative resource for anti-MRSA drugs [133]. Apart from that, sea cucumber also possesses antioxidant property that can scavenge free radicals, whereby the presence of excessive free radicals is associated with impaired wound healing. The antioxidant activity of sea cucumber species; *Bohadschia mamorata vitiensis, S. variegatus* Semper, and *S. badionotus* Selenka might be contributed by the content of coelomic fluid, as reported by Hawa et al. (1999) [139]. Additionally, Althunibat et al. (2009) reported high antioxidant activities of three sea cucumber species, *Holothuria scabra, Holothuria leucospilota* and *S. chloronotus* at 77.46%, 64.03% and 80.58% respectively, against linoleic acid free radical [129]. In addition, the antioxidant activities of Atlantic sea cucumber, *Cucumaria frondosa*, might be attributed to its phenolic content, particularly flavonoids which act against oxidative reactions [118]. However, studies on the antioxidant effects of sea cucumber on wound healing are still lacking. Previous studies had focused only on the anti-inflammatory and antibacterial activities of sea cucumber on wound healing [127,133,136]. Thus, to elucidate the antioxidant property of sea cucumber on wound healing, further studies should be performed. Additionally, other biological activities of sea cucumbers on wound healing should be explored. However, harvesting sea cucumber for extraction is not sustainable. Therefore, identification of active compounds and upscaling the production are needed.

## 6. The Advancements of Delivery System for the Selected Natural Products

There are several advancements in the delivery system which maximize the therapeutic benefits of natural products, including hydrogel, scaffolds, conjugates, hydrocolloids, foam, nano- and micro-particles [140,141]. This review focused on hydrogel, particles, polymeric bandage, and conjugates. These delivery systems have different mechanisms of action in promoting wound healing properties. For instance, hydrogels, which are the water-based gels have a gentle and effective debriding and desloughing actions by rehydrating necrotic tissue and removing it without damaging nearby healthy tissues [141]. Conjugation with biomaterials that possess good viscoelastic, hydrophilic properties and lower molecular weight such as hyaluronic acid (HA) have been reported to stimulate angiogenesis, induce inflammation, regulate cell adhesion and promote proliferation of keratinocytes and fibroblasts [34]. In addition, the use of particles (micro or nano) may provide slow and sustained release of active compounds to wounds, enabling reduction in dose and frequency of dressing on wound.

## 7. Conclusions

Successful wound healing is important for the quality of life of the patients. This review highlighted several natural products with bactericidal, immunomodulating and wound healing activities. These products are derived from floral and faunal sources commonly found in the tropical regions. Most of these agents have been used since antiquity for wound healing. Although many in vivo and in vitro studies have proven their effectiveness in wound healing, documented clinical trials are still lacking and need to be conducted more to ensure the safety and effectiveness in human application. The current challenge lies on identification of the active compounds responsible for their wound healing properties and the mechanism involved. Upscaling the production of these compounds needs to be studied as the demand of wound healing agents is high. Management of wound contracture, exudates and bacterial control are still the major challenges in wound healing. Wound contraction is a natural mechanism for the closure of open wound in healing process; however, excessive wound contraction leads to physical deformity, functional limitations and disability. Most of the wound dressings do not possess the capabilities to absorb thick and viscous exudates from wound. With the advent of multi-resistant bacteria, agents to enhance wound healing and prevent breaching of bacteria to the body system are also particularly relevant. Therefore, further investigations could focus on evaluating the ability of natural products in wound contraction control, exudates management and prevention of infections effectively at the wound sites.

## Figures and Tables

**Table 1:** The effects of curcumin on wound healing in animal studies.

Wound Type	Animal Type	Intervention, Dose & Route	Findings	Study
Incisional wounds	C57BL/6J male mice	Curcumin (0.2 mg/mL) in Pluronic F127 hydrogel (20%), topical	Fast wound closure with well-formed granulation tissue dominated by collagen deposition and regenerating epithelium.	[25]
	Male Sprague-Dawley rats induced with dexamethasone intramuscularly	Curcumin with 0.1% ointment in polyethylene glycol base, topical	Curcumin significantly accelerated healing of wounds by reductions in wound width and gap length compared to controls. Curcumin treatment resulted in enhanced expression of TGF-β1 and TGF-β tIIrc in both normal and impaired healing by immunohistochemistry assessment. Macrophages in the wound bed showed an enhanced expression of TGF-β1 mRNA in curcumin-treated wounds as evidenced by in situ hybridization.	[26]
	Male Sprague-Dawley rats	Curcumin/gelatin-blended nanofibrous mats (NMs), topical	The wounds treated with NMs showed regenerative process via (a) mobilization of wound site fibroblasts by activation of the Wnt signaling pathway, partly mediated through Dickkopf-related protein-1; (b) persistent inhibition of the inflammatory response through decreased expression of monocyte chemoattractant protein-1 by fibroblasts.	[35]
Excisional wounds	Male Wistar rats	2% curcumin, topical	Incomplete but normal healing at 21 days of treatment	[29]
		2%, 3%, and 5% THC, topical	For 2% THC, progressive but incomplete healing was observed at 21 days of treatment	
		2%, 3%, and 5% glucosyl-THC, topical	For 2% glucosyl-THC, complete healing of wounds at 21 days of treatment	
	Sprague-Dawley male rats	Curcumin loaded oleic acid based polymeric (COP) bandage, topical	Increased wound reduction and enhanced cell proliferation in COP bandage-treated groups. Comparative acceleration in wound healing. Western blotting and semi quantitative PCR analysis clearly indicate that COP bandage can efficiently quench free radicals.	[32]
	Streptozotocin-induced diabetic Swiss albino mice	Curcumin conjugated to hyaluronic acid (HA) (HA-Cur), topical	HA-Cur treated group showed well-formed granulation tissue rich in fibroblast, collagen and re-epithelialization was almost complete.	[34]
	Whole-body γ irradiated Swiss albino mice	Curcumin suspended in 0.5% carboxy methylcellulose (CMC), 100 mg/kg orally before irradiation	Pre-treatment with curcumin significantly enhanced the rate of wound contraction, decreased mean wound healing time, increased synthesis of collagen, hexosamine, DNA, and nitric oxide and improved fibroblast and vascular densities.	[36]
	Streptozotocin-induced diabetic male Wistar rats	400 μL of curcumin (0.3%) in PF-127 hydrogel (25%), topical	Curcumin increased the wound contraction, levels of anti-inflammatory cytokine (IL-10), antioxidant enzymes (SOD, CAT and GPx) and decreased expressions of inflammatory cytokines/enzymes (TNF-α, IL-1β and MMP-9). Histopathologically, the curcumin-treated wounds showed better granulation tissue dominated by marked fibroblast proliferation and collagen deposition; wounds were covered by thick regenerated epithelial layer.	[37]

**Table 2:** The effects of vitamin E on wound healing in animal studies.

Wound Type	Animal Type	Intervention, Dose & Route	Findings	Study
Excisional wounds	STZ-induced diabetic rats	α-tocopherol or PVE (200 mg/kg, oral)	Wound area was smaller at day 10; total protein content, collagen content SOD, and GPx activities were increased; MDA level was reduced; epidermis was well-formed and differentiated; re-epithelialization was completed; collagen was well-aligned.	[52]
	STZ-induced diabetic rats	Tocopherol (0.1 g, topical)	Wound closure rate was increased; total protein content and collagen fibers were increased; epithelialization was completed; epithelium was well-formed; interdigitation between epithelium and dermis layers was increased.	[51]
	Alloxan-induced diabetic mice	γ-tocopherol (35 mg/kg, oral)	Wound closure rate was accelerated; levels of 4-HNE, NF-κB, TNF-α, & IL-1β were reduced; CAT level was increased.	[53]
Excisional wounds inoculated with MRSA	BALB/c mice	Vitamin E (60 mg/kg, oral) + tigecycline (2 mg/kg, i.p.) or daptomycin (7 mg/kg, i.p.)	Bacterial load was reduced; natural killer cell cytotoxicity and leukocyte populations were increased.	[67]
		Annatto tocotrienol (50 or 100 mg/kg, oral) + daptomycin (7 mg/kg, i.p.)	Bacterial load was reduced; natural killer cell cytotoxicity and wound repair markers were increased.	[66]
Incisional wounds	Sprague-Dawley rats	Copper-doped borate bioactive glass/poly(lactic-co-glycolic acid) dressing loaded with vitamin E (3%, topical)	Wound closure rate was faster; number and density of new vessels were increased; new endothelium formed was longer; collagen deposition was thicker.	[57]
	Wistar rats	Medium chain triglycerides + linoleic acid + vitamin A, vitamin E + soy lecithin (0.1435 g, topical)	No acceleration in the process of tissue repair	[59]
	STZ-induced diabetic rats	α-tocopherol (200 mg/kg, oral)	Rate of wound closure was accelerated; SOD, GPx, and CAT activities were increased; MDA level was reduced	[54]
	Diabetic female C57BL/KsJ db+/db+ mice	Raxofelast, a synthetic analogue of vitamin E (15 mg/kg, i.p.)	Impaired wound healing was improved; breaking strength and collagen content of wound were higher; MDA level and MPO activity were decreased; re-epithelialization was moderate to complete; granulation tissue was well-organized; newly formed capillary vessels were observed; degree of infiltrated inflammatory cells was minimal.	[58]
	Diabetic C57BL/KsJm/*Leptdb* (*db*/*db*) mice	Mono-epoxy-tocotrienol-α (MeT3α) (1 μmol)	Closure of wound area was enhanced.	[55]
Skin irradiated with argon and copper-vapor laser	Yorkshire pig	Vitamin E (5 mg/kg, i.m.; 2 mg/cm^2^, topical)	Healing time for laser injury was reduced.	[56]

**Table 3:** The effects of honey on wound healing in animal studies.

Wound Type	Animal Type	Intervention, Dose & Route	Findings	Study
Excisional wounds	Sprague-Dawley rats	Gelam honey (topical)	Wound healed earlier; wounds exhibited less scab; formation of thin scar.	[71]
		Honey (topical)	Wound healed in 21 days; collagen deposition and neovascularization was increased	[72]
		Pectin-honey hydrogel or Manuka honey (topical)	Wound area reduction rate was faster; entire surface of lesion was covered with new epithelium; dermis was well-developed; mature fibrous tissue proliferation was observed.	[93]
		*Teucrium polium* honey (topical)	Healed wound area percentage was increased; re-epithelialization, granulation tissue formation, collagen arrangement, fibrinoleukocytic exudates severity, vasculature hyperemia or congestion, dermis organization and epidermal appendage reappearance were improved	[94]
	Swiss Wistar rats	Acacia honey (5% or 10%, topical)	Period of epithelialization was reduced; wound contraction was increased.	[95]
	Albino N-Mary Rats	Natural honey (10 mL) + zinc sulphate (36.3 mg) (topical)	Tensile strength was increased; collagen fibers, re-epithelialization and re-vascularization were increased.	[96]
	*Oryctolagus cuniculus* rabbits	Natural honey (topical)	Tensile strength of wounds, wound contraction, neovascularization, fibroplasias, epithelialization, and formation of collagen were increased	[97]
	New Zealand White rabbits	Honey (chestnut, blossom, or rhododendron) (topical)	Formation of granulation tissue, epithelialization, angiogenesis, and fibroplasia levels were increased on day 7.	[98]
	BALB/cCrSlc mice	Indonesia pure honey (0.1 mL, topical) or Manuka honey (0.1 mL, topical)	Wound area was smaller; myofibroblasts and new blood capillaries were observed on day 3	[71]
		Japanese honey (Acacia, Buckwheat flour, or Chinese milk vetch) (0.1 mL, topical)	Wound size was reduced in inflammatory phase, increased in proliferative phase and then decreased in proliferative phase; some wounds were not completely covered with new epithelium.	[74]
		Japanese honey (Manuka, Acacia, Chinese milk vetch) (0.1 mL) + hydrocolloid dressing (topical)	Wound area was larger; numerous macrophages were observed; no significant difference in TNF-α and TGF-β levels; re-epithelialization and collagen deposition were delayed.	[75]
Excisional wounds inoculated with MRSA	Wistar rats	*Melipona scutellaris* honey (0.1 mL/cm^2^, topical)	Bacterial count was decreased; levels of TNF-α, IL-1β, and IL-6 were increased; density of healing parameters (collagen, leukocytes and fibroblasts) were higher.	[99]
Incisional wounds	Standardbred horses	Manuka honey (2 mL, topical)	Wound area was smaller; wounds healed faster.	[100]
	Sprague-Dawley rats	*Teucrium polium* honey (topical)	Healed wound area percentage was increased; re-epithelialization, granulation tissue formation, collagen arrangement, fibrinoleukocytic exudates severity, vasculature hyperemia or congestion, dermis organization and epidermal appendage reappearance were improved	[94]
	Swiss Wistar rats	Acacia honey (5% or 10%, topical)	Breaking strength was increased.	[95]
	Wistar rats	Honey (topical)	Wound area was decreased; there were more collagen fibers and fibroblasts in honey-treated group.	[101]
		Honey + propolis (topical)	Wound area was decreased; number of vessels, collagen, fibroblast and cellular activity were increased.	[102]
Burn wounds	Wistar rats	Honey, milk, and *Aloe vera* ointment (5%, topical)	Wound area was decreased; thickness of the epidermis, fibroblast, and blood vessel counts were increased; scar tissue was softer.	[103]
	Swiss Wistar rats	Acacia honey (5% or 10%, topical)	Period of epithelialization was reduced; wound contraction was increased.	[95]
	Guinea pigs	Ulmo (*Eucryphia cordifolia*) honey (topical)	Eschar was shed earlier and diameter of wound was smaller at day 6–7; diameter of wound decreased to 50% at day 10; no epidermal regeneration was observed; an initial proliferative stage was observed with abundant cellularity, active fibroblasts and neoformation of blood vessels in the superficial dermis.	[104]
		Ulmo (*Eucryphia cordifolia*) honey + ascorbic acid (topical)	Eschar was shed earlier and diameter of wound was smaller at day 6; diameter of wound decreased to 50% at day 10; epidermis was generated; an advanced proliferative stage was observed with numerous blood vessels and small capillaries in the superficial dermis, the scar tissue zone presented a fibroblastic reaction, proliferation of collagen fibers, dense connective tissue, and thin collagen fibers.	
Burn wounds inoculated with *P. aeruginosa*, *K. pneumonia*, or *A. baumannii*	Sprague-Dawley rats	Tualang honey (0.1 mL/cm^2^, topical)	Wound size and bacterial count were reduced in *P. aeruginosa*-inoculated wounds; wounds healed completely on day 21 in *P. aeruginosa*- and *A. baumannii*-inoculated groups; no statistically significant anti-bacterial effects in *K. pneumonia*-infected wounds.	[73,105]

**Table 4:** The effects of sea cucumber on wound healing in animal studies.

Wound Type	Animal Type	Intervention: Species, Route	Findings	Study
Excisional wounds	Female Sprague Dawley rats	Total sulphated glycosaminoglycan from *S. hermanni* and *S. Vastus*, topical	The contraction rate of wounds treated with total glycosaminoglycan from *S. hermanni* and *S. Vastus* showed significant (*p* < 0.05) improvement compared to control group	[124]
	Either gender of Albino Wistar rats	*S. horrens* processed oil extract twice daily for consecutive 21 days, topical	At day 4, *S. horrens* treated group exhibited significantly smaller wounds compared to the negative control (*p* < 0.001) On days 8, 12, 16 and 21; *S. horrens* treated group and controls showed no significant change in the wound contraction.	[125]
	Male Sprague Dawley rats	*S. chloronotus* at 0.1%, 0.5%, and 1.0% *w*/*w* mixed with emulsifying ointment once a day for 10 days, topical	*S. chloronotus* treated group demonstrated a reduction in wound more rapidly compared to other groups starting on day 6. On the 6th post-wound day, *S. chloronotus* 0.5% demonstrated a significant wound reduction compared to all other groups (*p* < 0.05).	[126]
Burn wounds	Male Sprague-Dawley rats	*S. hermanii* Hydrogel dressing followed by OpSite^®^ film dressing as secondary dressing, Topical	At days 7, 14; No significant differences were observed in the measurement of wound contraction between all experimental groups. At days 21, 28; *gamat* hydrogel significantly increased wound contraction rates. The mRNA levels for pro-inflammatory cytokines (IL-1a, IL-1b, and IL-6) in wounds treated with *gamat* hydrogel were maintained at low levels throughout the healing process as compared to other groups. Histologically, advanced healing was observed; early re-epithelialization with proliferating and migrating keratinocytes projected toward the center of the wound.	[127]
Heat-burn wound inoculated with MRSA	Male Sprague-Dawley rats	30 μg/mL *S. badionotus* methanol extract applied topically twice daily.	The wound reduction measurement of *S. badionotus* showed no significant different with vancomycin.	[133]
Traumatic ulcer induced with heated burnisher	Male Wistar rats.	20%, 40% and 80% of *S. hermanii* prepared as gel with polyethylene glycol (PEG) 400 and 4000 solvent, applied one time to ulcer (0.1 mg)	40% *S. hermanii* treated group showed the biggest number of lymphocytes, while the 80% *S. hermanii* extract showed decreased number of lymphocytes.	[134]
